# Opioid‐related policy changes: Experiences and perspectives from people who use opioids to manage non‐cancer chronic pain

**DOI:** 10.1111/dar.13683

**Published:** 2023-05-30

**Authors:** Sarah Haines, Michael Savic, Suzanne Nielsen, Adrian Carter

**Affiliations:** ^1^ Turner Institute for Brain and Mental Health Monash University Melbourne Australia; ^2^ Turning Point, Eastern Health Melbourne Australia; ^3^ Monash Addiction Research Centre Monash University Melbourne Australia

**Keywords:** analgesics, chronic pain, opioid, public health

## Abstract

**Introduction:**

People who use prescription opioids to manage non‐cancer chronic pain are particularly vulnerable to opioid‐related policy change. This study aims to better understand what prescription opioids provide this population, what concerns they have in the context of new and changing opioid policies, such as the recently implemented prescription drug monitoring program in Victoria, Australia, their experiences of prescription opioid use, chronic pain and what they would like their healthcare to look like.

**Methods:**

Semi‐structured interviews were conducted with 30 people who use opioids to manage chronic non‐cancer pain.

**Results:**

Prescription opioids played an important role in supporting quality of life and mental health. However, experiences of stigma and lack of empathy from healthcare providers were common. Participants sought accurate information about their medications and expressed a desire for shared decision‐making in healthcare.

**Discussion and Conclusion:**

Prescription opioids can play an important role in pain management as well as social and psychological functioning for people living with non‐cancer chronic pain. Opioid‐related policy changes to medication availability need to consider the potential impacts that reducing, limiting or discontinuing opioids may have on this population. Including the voices of people who use prescription opioids to manage non‐cancer chronic pain in respectful, compassionate and meaningful ways.

## 1 INTRODUCTION

In Australia, prescription opioids are the leading cause of drug‐related death, with more people dying from prescription opioid overdoses than from road accidents [1]. While synthetic opioids are associated with the highest death rate in the USA, prescription opioids are still associated with significant harms, with 17,000 deaths attributed to their use in 2020 [2, 3].

Best practise guidelines for the treatment of chronic non‐cancer pain (CNCP) recommend a multidisciplinary approach that is tailored to the individual given the idiosyncratic mechanisms and dynamic consequences of biological, psychological and social factors that influence pain experiences [4]. Although multidisciplinary approaches to pain management and engagement with specialist pain clinics have been associated with improvements in pain and functioning for some individuals [5, 6], an evaluation of 15 systematic reviews assessing the effectiveness of psychological, physiological, complementary and alternative treatments for pain found that evidence was either limited, mixed or had small effect sizes [7]. Those who may benefit from multidisciplinary pain management and specialist pain clinics must first overcome long‐standing barriers such as cost, time, location and extensively long waitlists [8, 9]. Given CNCP is significantly more prevalent in individuals from lower socioeconomic backgrounds or who live in rural areas, these barriers are considerable [10].

People who use opioids to manage their pain condition face many risks. Addiction, overdose and death are well‐established concerns with the long‐term use of prescription opioids. Lesser‐known risks include cognitive decline, hyperalgesia (increased sensitivity to feeling pain), immunosuppression, osteoporosis, sexual dysfunction, sleep apnoea and increased risk of myocardial infarction [11]. These harmful side effects and tolerance to dose can limit the effectiveness of opioid use for CNCP and are no longer recommended as a first‐line treatment by most practice guidelines [12]. The benefits of opioid cessation in the context of pain severity, functioning and quality of life appear to depend upon the individual, with some people benefiting from discontinuation, while others experience no change or are worse off [13]. However, for CNCP associated with nervous and non‐nervous tissue injury, opioids remain one of the more efficacious pain relief option in the short and intermediate terms and have been associated with improvement in physical functioning and quality of life [7, 14].

People who live with CNCP are, therefore, highly vulnerable to public health policies that may limit their access to medication. These policies and responses have included restrictions on the purchasing of over the counter codeine, and the widespread implementation of modern prescription drug monitoring programs (PDMP) that seek to identify patients that may be at risk of opioid‐related harms. While such opioid‐related responses can prevent harms, they can also have negative impacts on people who use opioids to manage CNCP, such as increased stigma from healthcare providers, involuntary tapering and difficulty accessing appropriate care as a result of their opioid use [15, 16, 17, 18]. This population experiences both pain‐related stigma—manifested as not being believed or being blamed for their condition—as well as the stigma of using a medication associated with addiction and extramedical use [16, 19]. The concurrent opioid epidemic in North America has had a significant influence in shaping attitudes towards opioids more broadly. Popular media reporting rarely differentiates between the harms associated with illicitly obtained opioids, such as heroin and fentanyl, and those associated with prescription opioids. This increases fear and confusion among healthcare professionals and the general public [20, 21]. Finding a balance between appropriate pain management while avoiding potential harms from prescription opioids is critical to patient care [22]. However, it is unclear whether opioid‐related policies, such as PDMP, have achieved this balance.

Popular rhetoric around prescription opioid‐related harms often leaves little room for consideration of the affordances or benefits opioids provide to those who rely on them to manage CNCP [23, 24, 25]. Affordance refers to the various possibilities that might be enabled when drugs (or other technologies or social structures) are used in different situations [26]. Rather than viewing drug effects as an inevitably harmful consequence of the biomedical properties of drugs, social science scholarship on drugs views these as also being shaped by the social and environmental contexts in which drug use occurs [27, 28]. However, limited research has been conducted on the various roles that opioids play in the lives of people who live with CNCP, their experiences of healthcare in the context of newly implemented opioid responses, such as PDMP in Victoria, Australia or their desires for the future. Indeed, in March 2020, Victoria was the first state in Australia to legally mandate the use of PDMP before prescribing or dispensing controlled medications [29]. However, to the best of our knowledge, this is the first study in Australia to explore the experiences of this population in the context of such a major opioid‐related policy change. Given that people who use opioids to manage CNCP remain key stakeholders of opioid‐related policies [4, 15, 16, 17, 18, 20], it is remiss not to consider their experiences and perspectives of opioid use to inform policy responses. This paper examines: (i) what prescription opioids afford people who live with non‐cancer chronic pain; (ii) what concerns they have in the context of opioid‐related policy changes in Victoria, Australia; and (iii) what people who use prescription opioids would like their healthcare to look like.

## 2 METHODS

We conducted a qualitative study of 30 individuals who had received a prescription opioid to manage CNCP, aged 18 years or over, and lived in Victoria, Australia. An interim analysis revealed that data saturation; the point at which no new themes are identified [30], was reached after 30 interviews. Participants were recruited through paid social media advertising (Facebook) and received a $30 department and grocery store gift voucher for their time. As illustrated in Table 1, participants had been living with pain for 15 years on average (SD = 13.24) and utilised a range of opioid medications.

**TABLE 1:** Participant demographics, opioid medication type and years in pain.

	*n*	%
Sex		
Female	16	53%
Male	14	46%
Age	40–80 years (*m* = 59.13, SD = 10.93)	
Years living with pain	2–50 years (*m* = 15.25, SD = 13.24)	
Opioid medication		
Hydromorphone	9	30%
Oxycodone	7	23%
Hydrocodone	6	20%
Morphine	4	13%
Codeine	4	13%
Location		
Metropolitan	17	56%
Regional	8	26%
Rural	5	16%

*Note*: Only main/current opioid medication was recorded in cases where participants had been prescribed >1 across time. [Correction added on 09 June 2023, after first online publication: “Oxymorphone” under Opioid medication has been corrected to “Hydromorphone” in Table 1].

Interviews were conducted between August and December 2020, audio‐recorded and ranged from 1 to 1.75 hours in duration. Participants were interviewed via video conference or telephone by the first author using a semi‐structured interview schedule agreed upon by the authors. The study was approved by Monash University Human Research and Ethics Committee (Project number: 21128).

### 2.1 Data analysis

Thematic analysis produced a rich description of the dataset and highlighted key emergent themes [31, 32]. Although interview questions were informed by a review of the literature, a largely inductive (i.e., data driven) approach was used to analyse the data. Audio recordings were transcribed using a professional transcription company. Transcripts were analysed using Braun and Clarkes thematic analysis approach [31] and interpretation of themes was developed through discussion between authors. Illustrative quotes are presented verbatim to exemplify themes. Pseudonyms are used to protect participant privacy.

## 3 RESULTS

Three key themes emerged from the data. (Theme 1) *Affordances of prescription opioids: quality of life and mental health*, (Theme 2) *Unpleasant clinical encounters: Stigma and desire for empathy* and (Theme 3) *Respecting patient knowledge and wishes: Informed use and patient input*. These themes were interconnected and together gave insight into the functional and meaningful role that prescription opioids can play in the lives of people who live with CNCP. While participants were invited to speak about their experiences in the context of Victoria's newly implemented PDMP, most discussed opioid policy in general and their recent clinical experiences. As will be illustrated throughout each of the themes, participants were worried about any policy change that might threaten their access to opioids and were less concerned/aware of the specificities of particular policies.

### 3.1 Theme 1: Affordances of prescription opioids: Quality of life and mental health

Prescription opioids enabled participants to continue to engage in meaningful activities that may have otherwise been threatened by unmanaged pain and that participants felt significantly enhanced their quality of life. Activities included gardening, working, playing with grandchildren, spending time with friends, exercising and activities of daily living, such as grocery shopping, laundry washing, cooking and cleaning:

‘With opioids I can get out, I can do my shopping, I can go for a walk. Opioids allow me to do the things I want to do’—Margaret, 76 years old.

‘Without the shackles of pain, I can spend time with friends and actually enjoy myself’ —John, 61 years old.

For many, the inability to engage in these activities stripped life of its meaning and pleasure:

‘If I can't do anything I want to do, what's the point?’—Shana, 42 years old.

Living with pain had significant implications for mental health, including feelings of hopelessness and helplessness, isolation and loneliness, low mood and irritability. Access to prescription opioid medication appeared to alleviate these feelings with the promise of relief from pain, if only temporarily:

‘It's not just physical, it's mental too, because you become hopeless when you're in so much pain’—Richard, 53 years old.

Living with unmanaged pain left participants feeling isolated and lonely. Being around others in a socially meaningful way felt difficult when pain resulted in feelings of irritability and frustration:

‘Pain is an incredibly isolating thing. When my pain flares up, I don't want to be around anyone and no one wants to be around me’—Sharon, 50 years old.

The knowledge that pain‐relief was readily available helped participants cope by reducing anxiety around unmanaged pain and bolstered participants feeling that they could exert some agency when the pain was heightened:

‘Just knowing it's in the cabinet, even if I don't take it, knowing it's there actually helps me to be able to cope with the pain better. It's not a completely helpless situation’—Sue, 70 years old.

Losing access to prescription opioid medication due to policy changes was a daunting prospect. Those who strongly relied on their prescription opioids for pain relief, reported suicidal thoughts in relation to the prospect of losing access to their medication:

‘I'm so scared that one day they won't give me medicine [opioids]. With each new policy, it's getting harder and harder. There wouldn't be a lot of point staying around on this planet if that happened’—Richard, 53 years old.

‘Of course I think about suicide. If you can't access the one thing that works for you … anyone with chronic non‐cancer pain would’*—* Helen, 60 years old.

These quotes illustrate the perceived importance of the quality of life and mental health affordances of prescription opioids. In the context of already challenging lives (as the next theme will further illustrate), some participants' fear of losing access to prescription opioids (and what it allowed) through policy changes was palpable.

### 3.2 Theme 2: Unpleasant clinical encounters: Stigma and the desire for empathy

Experiences of stigma when trying to access opioid medications were common. Participants often became emotional recalling interactions with healthcare professionals in which they felt diminished based on their prescription opioid use. Stigma was characterised by experiences of not being believed by healthcare providers, being labelled an ‘addict’ and being made to feel professionally risky to provide care for due to liability issues surrounding their opioid medication use:

‘Health care providers fear people who use prescription opioids. They think you're lying, or you're a junkie and that they'll lose their [registration] if they have anything to do with you. We're just a liability to them, not a person in need of their support’*—* Jim, 40 years old.

Participants' experiences of stigma were evident in clinicians' body language, including their tone of voice, lack of eye contact or use of short sentences:

Facilitator: ‘You said that the GP [general practitioner] made you feel like a “junkie”. Can you explain what gave you that sense?’

‘Hmmm, it's sometimes subtle but you can feel it. I guess it's the tone of voice, the words that came out of his mouth—“**you** have a **problem**”. That distrustful look. It makes you feel like a second‐class citizen’*—* Maggie, 61 years old.

Participants understood that the PDMP was intended to safeguard them from opioid‐related harms; however, the policy was not viewed as entirely benevolent or without unwanted side effects:

‘I don't want to die from my medication. It's good in the sense that the government is looking out for us, but by the same token, I don't want to be treated like rubbish and left to rot in pain as a result!’—Melisa, 70 years old.

Participants desired increased empathy and understanding from their healthcare provider when making decisions about opioid medication supply. However, many reported feeling a distinct lack of empathy from their treating healthcare provider. This lack of empathy was attributed to multiple factors, including healthcare providers' inability to feel or see the pain, having exhausted their pain management options or not having the time to listen and engage:

‘Walk a mile in my shoes, then tell me you're going to stop the meds’—Linda, 70 years old.

Participants felt that greater empathy from their treating healthcare provider would significantly improve their clinical interactions by making them feel understood, even if the healthcare provider could not necessarily help with the pain:

‘Just by showing me that you're trying to understand helps, it helps a lot’—Linda, 70 years old.

As will be discussed in the next theme, participants reflections on improving care also coalesced around respecting patient knowledge.

### 3.3 Theme 3: Respecting patient knowledge and wishes: empowered self‐care and patient input

#### 3.3.1 Empowered self‐care

Participants empowered themselves by learning about their medication and using this knowledge to reduce risk where possible. They sought accurate information about the medications they were taking, including the effectiveness (or lack thereof) and the risks of prolonged use, including addiction and respiratory depression:

‘I keep very up to date and read widely about opioids. They don't actually fix pain, just mask it. They're a crappy drug but they're the only thing that seems to work’*—* Anita, 45 years old.

Despite these risks, many felt the benefits of pain relief in the absence of alternatives were worth the risks. Participants engaged in practises to reduce their risk by intermittently decreasing their dose, only taking their medication during a pain flare up or avoiding medication use at night:

‘If I ever feel I'm taking a few too many, a bit too regularly, then I will make a concerted effort to lay off for a bit’—Bill, 56 years old.

‘The trick is to save them for when you really need them. They're more effective that way and you don't get hooked’—Julie, 50 years old.

‘I take them at least 5 hours before bed so I don't impact my breathing at night’—Ian, 67 years old.

For some, prescription opioid use was conceptualised as valuing quality of life over quantity, even in the case of early death:

‘I would prefer to have five good years of pain management and die from an overdose, then live a life in pain without opioids’—John, 61 years old.

While quality of life was supported by opioids in some domains (as discussed above), the medications significant side effects appeared to offset this benefit somewhat. All participants acknowledged that opioid use came with unwanted and unpleasant side effects that negatively impacted them, including constipation, mental fogginess, feeling ‘zoned out’ and balance difficulties. Participants wished that there were effective alternatives to manage their pain, however, had found nothing that worked for them as opioids did. To manage the side effects, participants factored them into how they engaged in activities:

‘If the medication is making me feel a bit foggy, I will use my gardening chair when pruning and weeding to make sure I don't tumble’—Joan, 70 years old.

‘If I know I'll be spending the afternoon with a friend, I'll rest during the morning and only take a pill once I know I'll be up and about’—Sharon, 50 years old.

#### 3.3.2 Desire for patient input

Despite having good insight into their medication and pain experiences, participants often felt left out of decisions relating to their healthcare and wanted greater agency. This feeling was characterised by being ‘told’ instead of ‘discussing’ pain management plans, not being invited to share their point of view, and not having their perspective about the risks and benefits of their medication use heard. This was especially true when the participant's use had been qualified as ‘high risk’ by the PDMP as Michaela and Bill experienced:

‘My doctor kinda just tells me how things are going to be. Not interested in how I feel or my thoughts at all’—Michaela, 49 years old.

‘What he sees as too risky, I see as a risk worth taking. Why does he get to decide when I'm the one that has to live with the decision?’—Bill 56 years old.

Shared decision making was also considered as a way to build a relationship with the healthcare provider. For participants, a good doctor–patient relationship reduced feelings of waiting room anxiety before an appointment and resulted in better outcomes due to their preferences and personal circumstances being considered:

‘I had a doctor once and we were like a team. We tried one thing, and if it didn't work we'd put our heads together and see what else we could try’—Tim, 45 years old.

‘You feel so much more relaxed before your appointment when you're on good terms with the doctor’—Edward, 60 years old.

As these quotes illustrate, patient input enabled participants to feel more respected, included in care and connected to their healthcare provider, and they desired more encounters like this in the future.

## 4 DISCUSSION

This study aimed to better understand how people who use prescription opioids to manage CNCP made sense of their medication, their experiences in healthcare, their concerns about opioid policy changes and their hopes for future clinical encounters. These findings provide guidance for policy makers and healthcare providers that have implemented or are considering implementing changes to opioid policy.

### 4.1 Opioids may support quality of life and mental health for some patients

This study found that prescription opioids can play an important social and psychological function for people living with CNCP. The medication afforded participation in physical and social activities that contributed to a sense of quality of life, as well as a protective factor against the helplessness and hopelessness that can accompany pain. Living with CNCP can significantly impact a person's mobility, with implications for engaging in both enjoyable activities as well as activities of daily living [10]. CNCP can lead to increased social isolation due to the significant disruption to personal and professional life [33]. In line with previous research, the pain relief provided by prescription opioids played an imperfect but critical role in helping participants live in a way they found meaningful and socially connected [23, 24, 25]. It is clear that long‐term opioid use can be dangerous and that, for some people, opioid use may increase pain while decreasing functioning and quality of life. However, changes to prescription opioid medication policy or availability need to consider the potential impacts that reducing, limiting or discontinuing opioids may have on patients whose quality of life and mental health are supported by opioid medications. Policy makers and healthcare providers should take the time to understand the role that prescription opioids are playing in the specific context of their patients’ lives and provide alternative ways to support them. Based on the findings from this study, failure to do so may result in patient isolation, low quality of life, poor mental health or suicide. In order to facilitate this, health systems need to be adequately resourced and equipped to enable access to alternative or adjunctive treatments and programs.

### 4.2 Stigma and lack of empathy impact this population

This study supports previous research that found policies and responses to opioids can perpetuate and facilitate stigma [3, 5, 34], with participants' own use of stigmatising language (e.g., ‘they think you're a junkie’) reflecting an intrenched stigma towards people who use opioids more broadly. Patients report feeling labelled as ‘addicts’ based on their use of prescription opioids [15]. PDMP supported clinical decision making is complicated by professional competence in managing ‘risk’ of opioid medications, personal beliefs and sociocultural attitudes towards drug dependence [35, 36, 37]. The outcome for the patient, whether it is support and treatment or discrimination and discontinuation of care, is heavily influenced by these factors. In the context of opioids, stigma can result in people avoiding seeking medical assistance [38] or disclosing concerns about their substances use [39], receiving poorer quality of care [40], reducing access to healthcare [41] or increasing their substance use due to guilt, blame or shame [42]. Patients have cited negative sociocultural attitudes and stigma as being more damaging for them than the harm caused by prescription opioids [23]. Health care providers adopting person‐centred language and care approaches when working with this population can help to avoid perpetrating stigma, and is consistent with the National Institute for Health and Care Excellence guidelines for the management of chronic pain in primary care [43].

Empathy in healthcare is the active effort to understand and respect patients' experiences and perspectives in a way that the patient can see [44]. We found that patients experienced a strong desire for healthcare providers to be more empathetic in the context of both their pain and their use of opioid medications to manage pain. Healthcare provider empathy has been associated with a 1–2 point reduction in patient's pain on a 10‐point visual analogue scale [45]. This arguably makes empathy more than a just ‘nice thing to do’ but an active mitigatable factor in patient's experience of pain and drug use. Policy experts and pain specialists agree that finding the balance between reducing harm while maintaining adequate pain management is a challenging dilemma. The findings from this study suggest that those who use prescription opioids in their daily lives are not naive to this dilemma and are in fact actively engaging with it on a daily basis and have a strong desire to be included in finding solutions together with their healthcare provider.

### 4.3 Patients to play an active role in their own healthcare

The findings from this study present a cohort of people who have a wealth of knowledge about the medication they use and express a strong desire to play an active role in the healthcare that they receive. Stevenson et al. [46] argued that meaningful shared decision making requires: (i) the establishment of an atmosphere in which a patient's views and opinions about their treatment are valued; (ii) treatment opinions discussed are compatible with patient values and lifestyle; (iii) information about options, risks and benefits is shared in a clear and unbiased manner; (iv) assisting patients to weigh up the benefits and risks of treatment; and (v) healthcare providers share treatment recommendations based on expertise while affirming patient preferences. In line with previous research, instances where the PDMP algorithm identified the patients’ medication use as ‘high risk’, little room appeared to be left for shared decision making in the form of discussion with the patient around their context or understanding of their own perceived risk status [47]. In cases where consumers are already not being listened to or invited to share their perspective or knowledge, there is a risk that this may be exacerbated by the presence of a PDMP‐generated alert.

Evidently, ethical and legal dilemmas arise in cases where the healthcare provider and patient do not agree, including; what constitutes quality of life and if this can this ever trump quantity of life, healthcare provider medical training knowledge and expertise versus patient knowledge of their body and pain, as well as level of risk each is willing to take on, with implications for prescriber reputation and liability [48]. The issue of prescriber liability is not a minor one. Although a patient may accept the risk of a medication, the prescriber is medicolegally responsible for the patient's safety. Prescribers risk losing their medical licence and practise insurance, as well as receiving significant fines if they are found to be prescribing in a pattern resulting in harm, including overdose or death [5, 49]. These issues are not easily addressed and depend heavily on the context of each case. However, one harm reduction strategy that was not raised by participants and could be useful to better balance out the issue of risk for both the prescriber and patient is the provision of take‐home naloxone (a rapid‐acting opioid antagonist), along with training for family members in cases where the patient does not live alone. The co‐prescribing of naloxone and opioids has been strongly advocated for by drug researchers and recommended by the Centre for Disease Control for at‐risk patients with chronic pain [50, 51, 52]. However, uptake of this has been minimal due to lack of awareness, unwillingness to prescribe due to association with illicit opioid overdose or fear of offending patients, incorrect assumption about opioid risk or patient reporting of actual opioid use [51, 53].

Nevertheless, this study demonstrates that people who use opioids to manage CNCP: (i) do not view these medications as a panacea; (ii) do not view them as a preferred option, but the only effective option; and (iii) are forced to choose between accepting high levels of opioid‐related risks or not being able to function in their daily lives. The US National Institute of Drug Addiction recognises that a key reason that people with CNCP pain face daily risks of overdose death or addiction is due to a lack of potent, non‐addictive and effective pain management alternatives. In response, the National Institute of Health set up a trans‐agency effort, *Helping to End Addiction Long‐Term* (known as the NIH‐HEAL initiative), to accelerate research to find scientific solutions to better understand and safely manage pain [54]. Although research in this area is promising, until effective alternative options are readily available to this population, they are left with the potentially challenging reality of using opioids to manage their condition. Healthcare providers working alongside people who use opioids to manage CNCP need to acknowledge this difficult reality and support this population with compassion and respect while the pain management evidence base is being built and beyond.

### 4.4 Limitations

While this study offers rich and important insight into the experiences and perspectives of people who live with CNCP, some important health considerations may have been absent from our analysis. For example, the intricacies of each participants health condition, including the presence of co‐existing illness or injury that may influence the type and quantity of medication and other retreatment they can receive. These results also reflect the views of a subset of people across Victoria, Australia, who responded to the online Facebook advertisement and may have been highly motivated by strong options or experiences to participate in the study. Further, the experiences of this region‐specific sample may not generalise to other regions with differing access to healthcare or medicines.

Finally, although policy makers and researchers (including ourselves) are often interested in the impacts of particular policies, our data suggest that people who use prescription opioids to manage CNCP may not be aware of the specificities of particular policies and/or may not be so concerned with isolating their effects. Instead, in a context where several policy changes have occurred (e.g., restrictions on over the counter codeine access, PDMP), people may perceive and experience any and all opioid‐related policy changes as inevitably threatening access to medication they value. In this scenario, a new policy like PDMP, may interact with previous restrictive policies, forming an environment/atmosphere/assemblage that is perceived as hostile towards opioids and the people that use them. Indeed there are very few, if not any, policies we can recall in recent years that have aimed to make it easier for people to access opioids [55, 56]. In this context, the specificities of a particular policy, like PDMP, may be overshadowed by the general policy environment. Given this, it may be useful for researchers and policy makers to more closely consider the experiences and impacts of the opioid policy environment, rather than evaluating or isolating any particular policy contained within.

## 5 CONCLUSION AND RECOMMENDATIONS

People who use prescription opioids to manage CNCP are highly vulnerable to opioid‐related policy changes, including PDMP. Experiences of stigma, both due to their pain and use of opioids were common for this population and reiterated the need for stigma reduction initiatives. Despite the risks, opioids afforded many patients greater quality of life and mental health. Participants sourced accurate information about their medication and were aware that opioids were not a panacea and that they carried risks. They strongly desired to play an active role in their own health care. The opioid and pain management crises are complex and not easily resolved. A significant amount of human and financial resources have been invested to address these issues. Engaging and including the voices of people who use opioids to manage CNCP in a respectful, compassionate and meaningful way in opioid‐related policy development and implementation is critical to the successful management of these issues. Recommendations for healthcare providers who encounter consumers with CNCP are explained in Box 1.

BOX 1 **Recommendations for healthcare providers who encounter consumers with chronic non‐cancer pain**

- Be aware that opioids may be playing an important role in providing quality of life and mental health for this population. Discuss this with consumers and enlist other services as appropriate to support them when considering reducing opioid medications.
- If continuing opioids is too risky, ensure an alternative accessible co‐created pain management plan is in place.
- In instances where opioids are being prescribed, discuss risk and harm reduction with the consumer, including the provision of take‐home naloxone.
- Understand that risks may be assessed based on different criteria for this population. This does not mean that the consumers assessment of risk should be held above medical experts; however, healthcare providers should make an effort to understand what is most important to the consumer and work together to optimise this goal as best they can.
- This population experiences high levels of pain and opioid‐related stigma. They may (understandably) be particularly sensitive to perceived judgement or lack of empathy. Healthcare providers should vocalise empathic statements so that the consumer is aware.
- Acknowledging the psychological impact that stigma has on the patient, providing patient‐centred care and expressing empathic statements are important to reduce stigma among patients and to promote a collaborative approach for patient care.

## AUTHOR CONTRIBUTIONS

Each author certifies that their contribution to this work meets the standards of the International Committee of Medical Journal Editors.

## FUNDING INFORMATION

Sarah Haines is funded by an Australian Government RTP Scholarship and Suzanne Nielsen is funded by an NHMRC Career Development Fellowship #1163961.

## CONFLICT OF INTEREST STATEMENT

Suzanne Nielsen has received untied research funding to study opioid harms from Seqirus and is a named investigator on an implementation study of buprenorphine depot funded by Indivior.
